# Enhanced Organic Fouling Control and Energy-Saving Strategies in PVDF Hollow Fiber Membrane Ultrafiltration via Intermittent Micro–Nanobubble Aeration

**DOI:** 10.3390/membranes16060182

**Published:** 2026-05-25

**Authors:** Zhaoyang Li, Xitong Wang, Nachael Mwanga, Jigao Fu, Weidong Gao, Jun Zhang

**Affiliations:** 1 College of Civil Engineering, Heilongjiang University, Xuefu Road 74, Nangang District, Harbin 150080, China; li01170@163.com (Z.L.); fujigao2001@163.com (J.F.); guiltysxul@gmail.com (W.G.); 2 College of Environmental Science and Engineering, Tongji University, Shanghai 200092, China; wangxitongg@163.com (X.W.); nachaelimwanga@gmail.com (N.M.)

**Keywords:** micro–nanobubble aeration, hollow fiber membrane, membrane fouling alleviation, energy saving

## Abstract

Micro-nanobubbles (MNBs) aeration has been widely reported as an effective approach for membrane fouling mitigation. However, their optimal operation in polymeric hollow fiber membrane (HFM) systems remains unclear. In this study, the antifouling performance of MNB-assisted ultrafiltration was systematically investigated using a PVDF-HFM, with particular emphasis on release pressure and intermittent aeration strategy. Increasing the release pressure to 0.60 MPa produced smaller and more concentrated bubbles, significantly alleviating membrane fouling. A distinct intermittent-aeration window was observed, in which a 15 min interval achieved the best overall performance, with a rejection efficiency of 75% and a cleaning efficiency of 93%, approaching that of continuous aeration. Longer intervals resulted in rapid deterioration in fouling control, indicating insufficient bubble replenishment. Compared with continuous operation, the optimized intermittent mode maintained comparable membrane performance while reducing energy consumption by approximately 50%, demonstrating a clear advantage in energy efficiency. Importantly, the optimal intermittent interval for PVDF-HFM (15 min) differs from that reported for ceramic membranes (30 min), highlighting that the performance of intermittent MNB aeration is not universal but strongly dependent on membrane properties. This shift in optimal interval is attributed to differences in surface wettability, structural flexibility, and local hydrodynamic conditions, which collectively influence bubble retention, interfacial shielding, and foulant detachment.

## 1. Introduction

Access to safe and clean drinking water is not only a basic human right but also a significant global challenge driven by population growth, rapid urbanization, and worsening environmental pollution. Among various water treatment technologies, ultrafiltration (UF) has emerged as a robust and efficient solution for removing suspended solids, bacteria, and macromolecules while maintaining a relatively low energy footprint compared to reverse osmosis [1]. However, the widespread application of UF is persistently hindered by membrane fouling, particularly organic fouling caused by natural organic matter (NOM) such as humic acid (HA) [2,3]. Organic foulants exhibit a high affinity for membrane surfaces, leading to the formation of dense gel layers or pore blockages, which significantly impair permeate flux and increase operational costs [4,5].

In contemporary water treatment, two major types of membranes are predominantly utilized: emerging ceramic membranes and well-established polymer hollow fiber membranes (HFM). While our previous studies and others have demonstrated the high durability and chemical resistance of ceramic membranes in fouling-heavy environments [6,7,8], hollow fiber membranes, typically composed of polyvinylidene fluoride (PVDF), remain the industry standard due to their high surface area-to-volume ratio, flexibility, and cost-effectiveness. Unlike rigid ceramic structures, the flexible nature of HFM allows for micro-vibrations under fluid stress, and its distinct surface hydrophobicity significantly influences the interaction between the membrane surface and foulants [9,10].

Chemical cleaning is widely used to restore membrane performance, but repeated exposure to oxidants, acids, and alkaline agents can accelerate polymeric membrane aging and shorten service life. This increases operating costs and generates additional end-of-life membrane waste [11]. Therefore, physical fouling-control strategies that reduce chemical cleaning demand are important for sustainable and circular membrane operation. In this context, micro–nanobubbles (MNBs) aeration offers a chemical-free or chemical-reducing approach for membrane fouling mitigation [12,13,14]. MNBs possess unique properties, including a high surface area-to-volume ratio, negative surface charge, and long residence time in water due to reduced buoyancy [15], and thus have extensive applications in the field of water treatment [16,17]. Previous studies have shown that their antifouling action is mainly associated with two physical mechanisms: turbulence-induced shear, which disrupts foulant accumulation on the membrane surface, and the gas-bridge effect, in which bubbles form a physical barrier between foulants and the membrane interface, thus suppressing direct deposition [18,19,20,21]. Furthermore, the high energy demand associated with continuous MNB generation remains a barrier to large-scale adoption. Intermittent release strategies have been proposed to balance performance and energy consumption [8].

While the efficacy of MNBs in assisting ceramic membrane filtration has been documented, there remains a critical knowledge gap regarding the optimization of MNB parameters—specifically release frequency and bubble concentration—for flexible HFM systems. This issue is scientifically important because the antifouling action of MNBs depends not only on bubble size and concentration but also on membrane surface properties and module hydrodynamics. Compared with rigid ceramic membranes, PVDF-HFM possess different surface wettability, flexible filament structures, and confined inter-fiber flow fields, which may alter bubble residence, interfacial coverage, foulant–membrane contact, and foulant detachment behavior.

Therefore, the novelty of this study lies in identifying and explaining the membrane-dependent nature of the optimal intermittent MNB aeration interval. We specifically evaluate the impact of release pressure and intermittent release patterns on HFM performance. Unlike previous studies that primarily focused on bubble characteristics or overall antifouling performance, this work seeks to elucidate how the optimal intermittent operation depends on membrane material and structure. By linking fouling behavior, separation performance, and fouling-layer characteristics, this study indicates that intermittent MNB operation should not be treated as a universal operating parameter, but should be optimized according to membrane material and configuration. This work will provide a mechanistic understanding of membrane-dependent MNB operation and offer guidance for the design of energy-efficient antifouling strategies in polymeric ultrafiltration systems.

## 2. Materials and Methods

### 2.1. Membranes and Chemicals

A commercial polyvinylidene fluoride (PVDF) HFM (Xinhui Membrane Technology Co., Ltd., Hangzhou, China) with a nominal pore size of 100 nm was used in this study. The membrane module had an effective filtration area of 0.0064 m^2^. The surface hydrophobicity of the PVDF membrane was characterized by a contact angle of 56.21°. Other properties and specifications are specified in Table 1. Before usage, the membranes were thoroughly rinsed with distilled water to remove any preservatives or residual contaminants.

Humic acid (HA), sourced from Sigma-Aldrich (Merck, Germany), was selected as a model foulant to simulate NOM fouling owing to its compositional similarity to natural organic matter, colloidal nature, and propensity to interact with membrane surfaces, thus offering a standardized approach for investigating and comparing fouling behavior. A concentrated stock solution (1000 mg L^−1^) was prepared by dissolving HA sodium salt in distilled water, followed by 60 min of intensive stirring. The feed water was subsequently prepared with a target HA concentration of 30 mg L^−1^, exhibiting a pH of 6.92–7.0 and a chemical oxygen demand (COD) of 42 mg L^−1^. Other parameters were adjusted as outlined in Table 2.

### 2.2. MNB Generation and Characterization

Micro and nanobubbles (MNBs) were generated using a hydrodynamic cavitation-based microbubble generator (model LF-02PT, Nano Scientific, Xingheng Technology, Shanghai, China). The system was operated by introducing air at a constant rate of 10 mL min^−1^ into a water stream flowing at 1300 mL min^−1^. The release pressure within the system was adjusted to 0.35, 0.50, and 0.60 MPa to regulate bubble size and concentration [21]. Bubble samples were collected immediately from the outlet of the MNB generator and analyzed without prolonged storage to minimize bubble dissolution and coalescence. The size and distribution of the generated microbubbles were determined using the laser diffraction technique with the Horiba LA-960 machine. The measurement of nanobubbles was conducted using the nanoparticle tracking analysis (NTA) technique with Zetaview (Particle Metrix, Inning am Ammersee, Germany). All measurements were conducted at room temperature (25 ± 1 °C), and each condition was measured in triplicate. The results are reported as mean values with standard deviations. More operation details have been described in our previous work [8].

### 2.3. Experimental Setup for HFM Filtration

**Figure 1:**
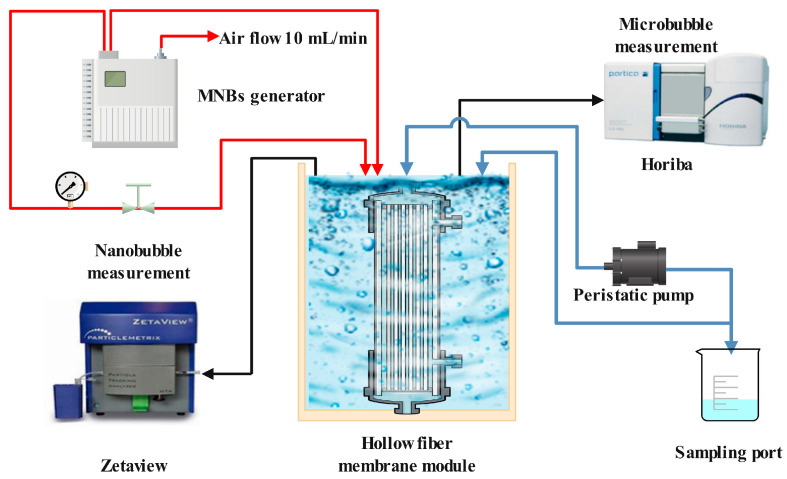
Experimental setup for HFM module coupled with MNB generation, measurement system. (Note: The red line represents the path of MNBs water, the blue line represents the membrane filtration path, and the black line represents the MNBs testing path).

The HFM filtration experiments were conducted in a dead-end mode using an 8 L feed container. A peristaltic vacuum pump (YZ1515X, Longer Precision Pump Co., Ltd., London, UK) was employed to maintain a constant permeate flux of 75 LMH. Transmembrane pressure (TMP) was continuously monitored and recorded using a digital pressure gauge (YB-80) at 30 min intervals. For MNB generation, feed water was extracted from the top of the container, while the generated MNB water was returned to the bottom of the container through a designated channel, as schematically shown in Figure 1. The filtration process lasted for 6 h, where the MNB generator release pressure was varied from 0.35 MPa to 0.60 MPa to optimize the size and concentrations of MNBs.

To evaluate the energy-saving potential, an intermittent MNB release pattern was introduced, where the generator was alternated between “on” and “off” states at specific intervals (10, 15, 30, 45, and 60 min). This scheme was designed to reduce MNB generation energy consumption by 50% compared to the continuous release mode. The alternation of on and off was repeated throughout the 6 h filtration cycle.

### 2.4. Analytical Method

The membrane performance was assessed based on three key metrics: fouling resistance, rejection efficiency, and cleaning recovery.

#### 2.4.1. Fouling Resistance

Fouling resistance was quantified by monitoring the normalized transmembrane pressure (n*TMP*) over a 6 h filtration cycle. Initial n*TMP* values were recorded, and subsequent measurements were taken at 30 min intervals throughout the process. Additionally, Scanning Electron Microscopy (SEM, ZEISS Gemini SEM 300, Oberkochen, Germany) and Energy-Dispersive X-ray Spectroscopy (EDS, OXFORD Xplore, Abingdon, UK) were used to analyze the cross-sectional morphology and elemental composition (specifically carbon deposition) of the fouled membrane surfaces.

#### 2.4.2. Rejection Efficiency

To evaluate the membrane’s ability to reject organic foulants, the concentrations in the feed and permeate were determined after 6 h of filtration. The rejection efficiency (Re) was calculated based on the HA concentrations in the feed (*C_f_*) and permeate (*C_p_*), determined by UV absorbance at 350 nm. UV350 refers to the UV absorbance of the feed water measured at 350 nm, which was used as an indicator of HA concentration. UV absorbance measurements were performed using the Hach spectrophotometry Model (Hach DR 6000, Loveland, CO, USA).

(1) Re=(1−CpCf)×100%

#### 2.4.3. Cleaning Efficiency

Following a 6 h filtration run, the membrane was backwashed using 1000 mL of MNB-enriched distilled water for 10 min at 0.0145 MPa. The cleaning efficiency was evaluated by calculating the recovery of n*TMP*:

(2) $$nTMP=\frac{TMP_{a}-TMP_{f}}{TMP_{0}-TMP_{f}}\times 100\%$$

where *TMP_a_* is the *TMP* after cleaning, *TMP_f_* is the final *TMP* before cleaning, and *TMP*_0_ is the *TMP* of the virgin membrane.

#### 2.4.4. Energy Consumption

The specific energy consumption associated with MNB generation was calculated according to Equation (3):

(3) $$E=\frac{P_{MNB}\times t_{on}}{V_{P}}$$

where *E* is the specific energy consumption for MNB generation (kWh m^−3^), *P_MNB_* is the electrical power of the MNB generator (kW), *t_on_* is the actual operating time of the generator during filtration (h), and *V_p_* is the permeate volume produced during the filtration cycle (m^3^).

## 3. Results

### 3.1. Screening of the Optimum MNB Condition for PVDF-HFM

**Figure 2:**
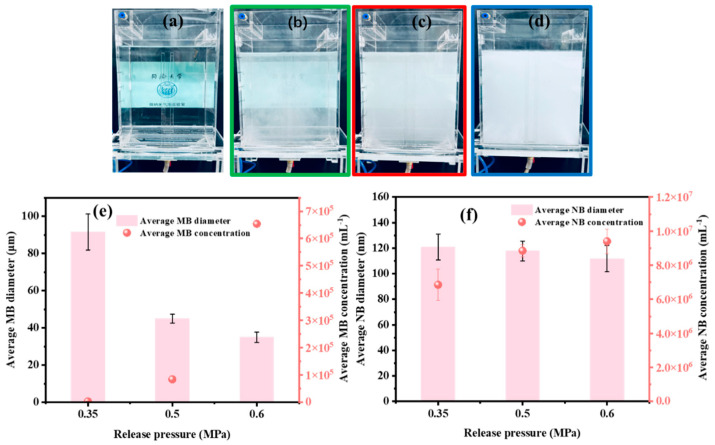
Effects of release pressure on microbubbles. (**a**–**d**) The appearance of water in the container before release, 0.35 MPa, 0.50 MPa, and 0.60 MPa, (**e**) average diameter and concentration of MBs against release pressure, (**f**) average diameter and concentration of NBs against MNB release pressure.

The release pressure of air-saturated water markedly affected the size and concentration of the generated MNBs. Initially, the water in the container was clear (Figure 2a), indicating the absence of microbubbles. However, as the release pressure increased from 0.35 to 0.6 MPa, noticeable changes occurred. The water became translucent (Figure 2b) and eventually turned into a milky white liquid (Figure 2c,d), indicating a high concentration of microbubbles. As the pressure increased from 0.35 to 0.60 MPa, the average microbubble diameter decreased from 91 to 31 μm, while the microbubble concentration increased from 3.27 × 10^3^ to 6.43 × 10^5^ particles mL^−1^ (Figure 2e). At the same time, nanobubble concentration also increased, from 6.85 × 10^6^ to 9.40 × 10^6^ particles mL^−1^, whereas the average nanobubble size remained relatively stable at approximately 120 nm (Figure 2f). These results indicate that increasing the release pressure favored the generation of smaller and more concentrated bubbles, which are expected to provide stronger membrane–bubble interactions during filtration. Accordingly, 0.60 MPa was selected as the optimum operating pressure for the subsequent intermittent release experiments.

**Figure 3:**
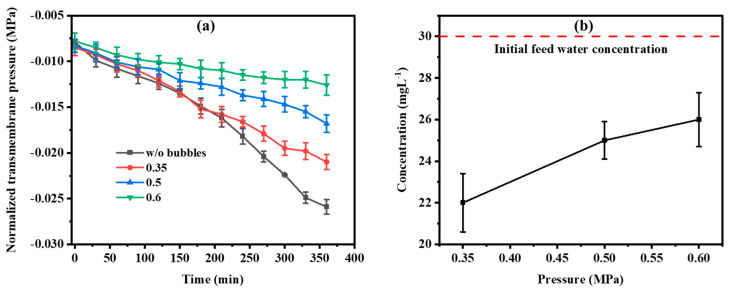
Illustrates the effects of release pressure on HFM fouling. (**a**) Normalized TMP of the membrane operated under different MNB release pressures plotted against time. (**b**) Feed water concentration after 6 h of filtration plotted against MNB release pressure.

The HFM antifouling performance with MNB aeration under different release pressure are shown in Figure 3, focusing on normalized TMP changes and changes in feed water concentration. To avoid relying solely on qualitative mechanistic interpretation, the relationship between MNB properties and fouling indicators was further compared. Increasing the release pressure from 0.35 to 0.60 MPa decreased the mean microbubble diameter and increased the microbubble and nanobubble concentration. The use of smaller and more concentrated bubbles substantially alleviated membrane fouling, reducing the final TMP from −0.021 MPa under larger and less concentrated bubble conditions to −0.0126 MPa under the smaller and more concentrated bubble condition. When assessing the feed water concentration after six hours, it became evident that small, highly concentrated bubbles resulted in a higher concentration in the water, peaking at 26 mg L^−1^. Conversely, the lowest concentration was observed with bubbles released at 0.3 MPa, reaching a minimum of 21 mg L^−1^ (Figure 3b). The higher residual HA concentration in the feed water at 0.60 MPa provides supporting evidence that less HA was deposited on the membrane surface during filtration. However, this indicator was interpreted together with TMP evolution and SEM/EDS observations rather than used as independent proof of reduced fouling. These results suggest that smaller and more concentrated bubbles reduced the direct deposition of HA on the membrane surface and maintained more HA in suspension. This pressure-dependent improvement can be attributed to the higher surface area, stronger collision probability, and longer effective action of small and concentrated bubbles, which collectively enhance foulant disruption and reduce foulant attachment to the membrane surface. It should be noted that the relationship among bubble size/concentration, fouling-layer morphology, resistance development, and rejection performance was not directly quantified under each MNB generation condition. Future studies should systematically decouple bubble size and concentration and combine TMP analysis, resistance-in-series modeling, SEM/EDS quantification, and foulant rejection measurements to establish a more quantitative bubble-property–fouling-mechanism relationship.

The antifouling effect of MNBs in the PVDF-HFM system can be attributed to the synergistic action of hydrodynamic disruption and interfacial shielding. Firstly, microbubbles induce localized turbulence and shear near the membrane surface, which disrupts foulant accumulation and limits the formation of a compact cake layer [22,23]. This effect is particularly important during the initial stages of fouling, where frequent bubble–foulant collisions hinder the transition from reversible deposition to irreversible attachment. Secondly, nanobubbles contribute to a gas-bridge effect by forming a discontinuous gaseous layer at the membrane–water interface, thereby reducing direct contact between hydrophobic foulants and the membrane surface [8,24]. This interfacial shielding weakens adhesion forces and suppresses pore blocking, especially for NOM foulants such as humic substances. In addition, the collapse and dissolution of MNBs generate micro-scale disturbances that further destabilize deposited foulants, promoting their detachment from the membrane surface. The combined action of these mechanisms leads to the formation of a looser and more reversible fouling layer, consistent with the reduced TMP development and enhanced cleaning recovery observed in the HFM system.

### 3.2. Intermittent Aeration Pattern for Fouling Control in PVDF-HFM

**Figure 4:**
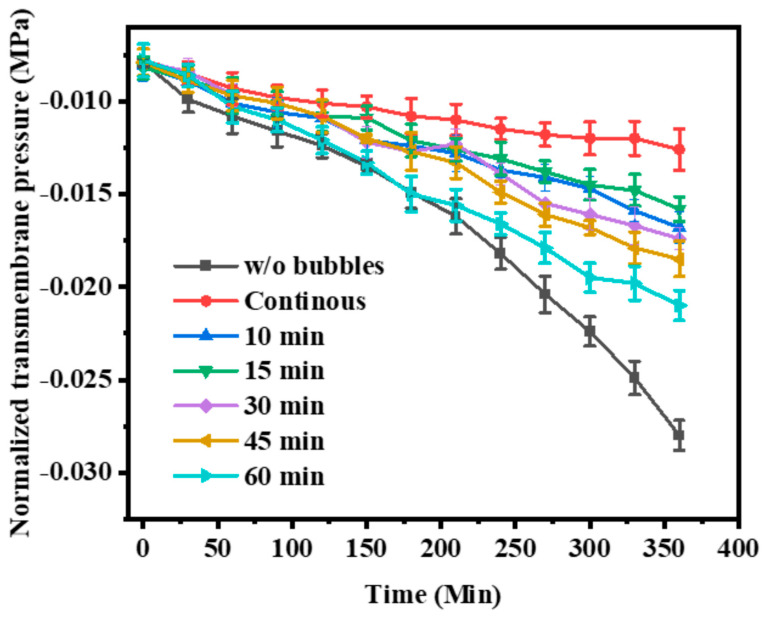
A plot of normalized TMP of membranes operated with various MNB release intervals against time.

Once the optimum bubble condition had been selected, the effect of intermittent release interval on HFM fouling was evaluated. Under intermittent MNB release, the fouling behavior of the HFM showed a clear interval dependence (Figure 4). The normalized TMP increased progressively as the release interval was extended, indicating that longer off-periods weakened fouling control. The most severe fouling occurred at 60 min, where the maximum normalized TMP reached −0.021 MPa. In contrast, the 15 min interval yielded a lower maximum normalized TMP of −0.0185 MPa and showed the closest behavior to continuous release among the intermittent modes examined. The gap between the optimum intermittent mode and continuous operation was small, indicating that properly timed bubble replenishment can retain most of the antifouling benefit without requiring uninterrupted aeration.

This behavior is consistent with the temporal evolution of MNB properties after generation ceased. According to our previous work [8], microbubble concentration dropped by more than 60% within 1 min, accompanied by an increase in mean bubble size from 32.5 to 45.9 μm, and the microbubbles disappeared within 5 min. Nanobubbles persisted longer, but their concentration declined with time and decreased sharply after 40 min. The deterioration in HFM performance at prolonged intervals therefore reflects progressive weakening of both the microbubble-driven turbulence effect and the nanobubble-associated gas-bridge effect. In practical terms, the HFM system requires relatively frequent bubble renewal to maintain effective membrane protection during filtration.

**Figure 5:**
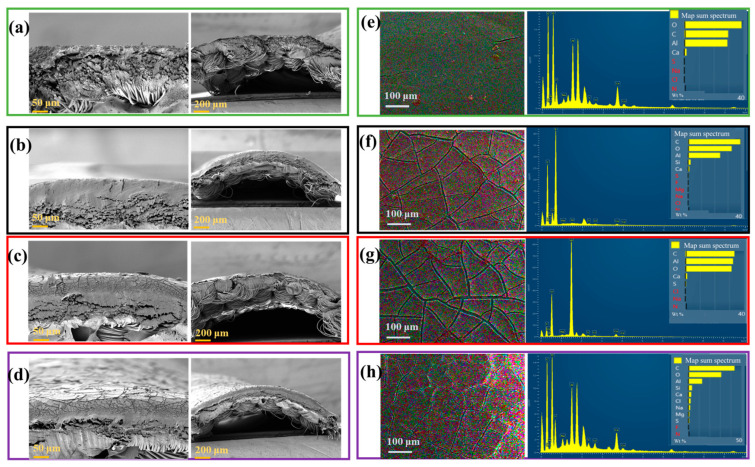
SEM images (**a**–**d**) and EDS results (**e**–**h**) showing the cross-sectional area of hollow fiber membranes. (**a**,**e**) New membrane. (**b**,**f**) Membrane operated without bubbles. (**c**,**g**) Membrane operated with continuous bubbles. (**d**,**h**) A 15 min interval bubble release operation.

The SEM analysis provided direct morphological evidence for the effect of MNBs on fouling-layer development over the PVDF-HFM. The virgin membrane exhibited a well-defined cross-sectional structure with no visible foulants and only a very thin active layer, indicating its intact initial morphology (Figure 5a). In contrast, the membrane operated without bubbles was covered by a dense foulant layer on the active side, demonstrating severe foulant accumulation under bubble-free conditions (Figure 5b). When MNBs were introduced, the foulant layer became less compact and exhibited numerous cracks, although a layer of similar overall thickness was still present (Figure 5c). Under the optimum intermittent MNB release condition, the membrane showed a morphology very similar to that observed under continuous bubbling, with a relatively loose and cracked foulant layer rather than a dense compact deposit (Figure 5d). These observations indicate that MNBs did not completely prevent foulant deposition, but significantly modified the deposited layer into a less compact and more weakly attached structure.

The EDS results further supported these morphological observations by revealing clear differences in foulant composition under different operating conditions. In the fouling layer, oxygen was the dominant element, followed by carbon, aluminum, and trace calcium (Figure 5e). However, in the absence of bubble release, the carbon signal increased substantially and even exceeded oxygen, indicating intensified deposition of organic matter on the membrane surface (Figure 5f). By contrast, membranes operated with continuous MNB release showed a noticeable reduction in carbon content (Figure 5g). Importantly, the optimum intermittent release condition displayed a similar decrease in carbon deposition to that of continuous bubbling, suggesting that optimized intermittent operation could suppress organic accumulation nearly as effectively as continuous release (Figure 5h).

### 3.3. Rejection Performance and Cleaning Recovery of PVDF-HFM

The HFM rejection results further confirmed the existence of an optimal intermittent release interval. Without bubble addition, the rejection efficiency was only 45%, indicating severe performance deterioration under organic fouling. Once intermittent MNB release was introduced, rejection improved substantially. The best intermittent result was obtained at 15 min, reaching 75%, which was only 3% lower than continuous bubbling. The 30 min interval still maintained a relatively high rejection of 73%, whereas longer intervals caused a marked decline, with rejection decreasing to 65% at 45 min and 59% at 60 min. These results demonstrate that the MNB-assisted HFM was highly sensitive to release frequency and that prolonged intervals could not sustain the same foulant-control effectiveness as shorter intervals.

**Figure 6:**
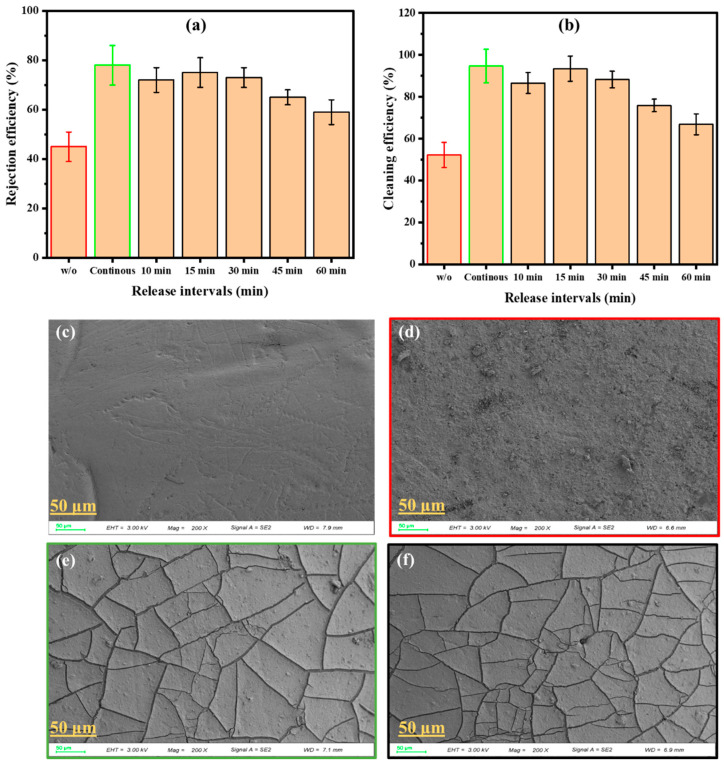
Illustrates the impact of release patterns on HFM rejection performance and cleaning recovery. (**a**) Rejection efficiency plotted against release patterns, and (**b**) cleaning efficiency plotted against release patterns. SME images of the (**c**) virgin membrane, (**d**) without bubbles, (**e**) continuous release and (**f**) 15 min interval release of MNBs’ operated HFM surface.

An even more pronounced advantage of the 15 min interval was observed in membrane cleanability. The optimal intermittent condition achieved a cleaning efficiency of 93%, only 1.5% lower than continuous release. The 30 min interval still showed strong recovery at 89%, followed by 86% at 10 min, whereas the 60 min interval declined sharply to 66%. This trend indicates that shorter MNB release intervals not only mitigated fouling during operation but also altered the deposited foulant layer in a way that made it easier to remove during backwashing. In other words, MNBs did not merely delay fouling formation; they increased the reversibility of the fouling layer. SEM images further supported these conclusions. In Figure 6c, clean membrane pores were visible, demonstrating effective cleaning. Conversely, without bubbles (Figure 6d), dense fouling covered pores, challenging backwashing and reducing cleaning efficiency. Interestingly, bubbles during operation (Figure 6e,f) led to a weaker fouling layer with cracks, easily cleaned through backwashing, improving efficiency. These results underscore the vital role of bubbles in enhancing cleaning processes and overall efficiency.

Taken together, the rejection and cleaning results suggest that 15 min represents the most favorable balance between bubble persistence and energy saving in the PVDF-HFM system. At this interval, enough bubbles were replenished to maintain effective membrane protection, yet the generator remained off for half of the operation time. This reduction in energy consumption suggests that implementing an intermittent release pattern could lead to considerable energy savings without compromising the effectiveness of the filtration process. This explains why the 15 min intermittent pattern could approach the performance of continuous bubbling while preserving the energy-saving advantage of intermittent operation.

### 3.4. Membrane-Dependent Optimum Interval: Why PVDF-HFM Differs from Ceramic Membrane?

A notable outcome of this study is that the optimum intermittent interval for PVDF-HFM differed from that previously established for the ceramic membrane system. In the earlier ceramic-membrane study, the best intermittent performance was obtained at 30 min, with only marginal differences from continuous release in normalized TMP, rejection efficiency, and cleaning recovery. By contrast, the present HFM dataset identified 15 min as the optimum interval, yielding 75% rejection and 93% cleaning efficiency. The optimum MNB release strategy is therefore not universal, but membrane-dependent.

**Figure 7:**
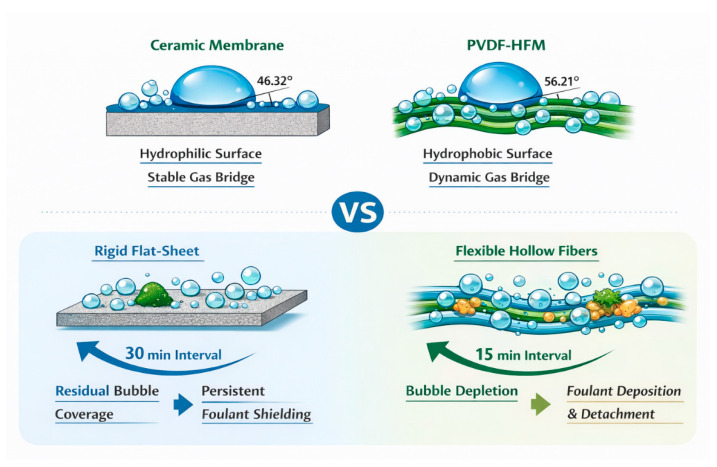
Membrane-dependent mechanisms governing optimal intermittent MNB aeration in ceramic and PVDF-HFM ultrafiltration.

Figure 7 show two explanations of MNBs mechanism for two types of membrane module. One plausible explanation is the difference in membrane surface properties. The PVDF-HFM here had a contact angle of 56.21°, whereas the ceramic membrane had a lower contact angle of 46.32°. Because one of the key antifouling mechanisms of MNBs is the gas-bridge effect, the more hydrophobic PVDF surface may favor stronger bubble attachment and more stable interfacial bubble coverage. At the same time, this protective layer may require more frequent replenishment in the hollow fiber environment, where the interface is more dynamic than on a rigid flat-sheet ceramic membrane. This interpretation is consistent with the experimentally observed shift in optimum interval from 30 min for ceramic membranes to 15 min for PVDF-HFM.

A second explanation lies in structural and hydrodynamic differences between the two membrane configurations. Hollow fibers are flexible porous filaments with confined local flow fields, whereas ceramic membranes are rigid planar structures. In the HFM module, bubble residence, collision frequency, and dissipation near the membrane are likely influenced by inter-fiber confinement and possible fiber micro-movements during operation. Under such conditions, longer off-periods would allow the protective bubble population to decay too far, resulting in renewed foulant deposition and stronger foulant attachment. The progressive increase in normalized TMP and the deterioration in rejection and cleaning performance at 45 and 60 min are consistent with this interpretation.

It should be noted that the 15 min optimum interval does not correspond to a distinct initial bubble size. All intermittent experiments were conducted at the same release pressure of 0.60 MPa; therefore, the initial bubble size and concentration were identical among different intermittent modes. The difference among the intervals was the extent to which the bubble population decayed during the off-period. Thus, the 15 min interval should be understood as the optimal bubble replenishment frequency under the selected 0.60 MPa generation condition. If the initial bubble size or concentration is changed, the optimal interval may also shift, further supporting the conclusion that intermittent MNB operation should be optimized according to both membrane properties and bubble characteristics.

### 3.5. Energy–Performance Trade-Off and Practical Implications

From a process perspective, the value of intermittent release lies in its ability to maintain membrane performance while reducing energy input. Based on the thesis calculation at 0.60 MPa, continuous release consumed 32 kWh m^−3^, whereas intermittent release reduced the energy demand to 16 kWh m^−3^, corresponding to a 50% reduction. This confirms that interval operation is not merely a conceptual optimization, but a practical strategy for lowering the energy penalty associated with MNB generation.

**Figure 8:**
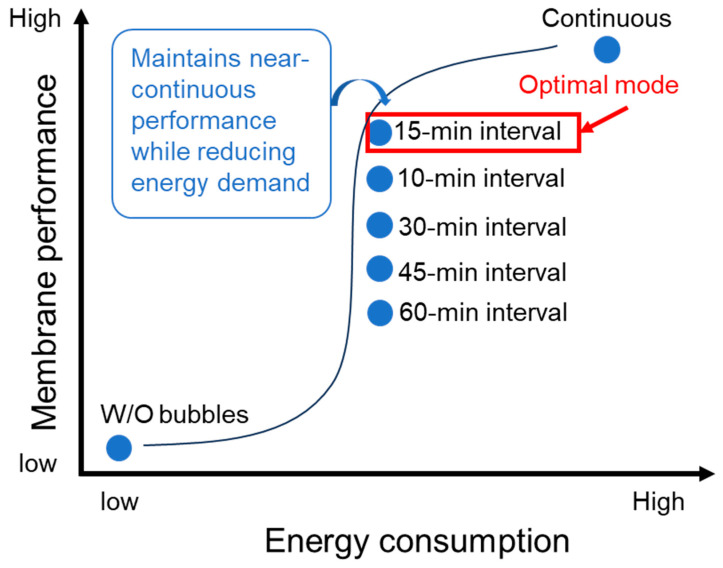
Energy–performance trade-off of intermittent MNB release in PVDF-HFM ultrafiltration.

When energy consumption is considered together with membrane performance, the trade-off effect becomes prominent. To more clearly illustrate this trade-off, the energy–performance relationship is summarized in Figure 8. Continuous MNB release provided the strongest fouling-control performance, but required uninterrupted generator operation. In contrast, the optimized 15 min interval maintained fouling alleviation, rejection efficiency and cleaning recovery close to those of continuous release while reducing half of energy consumption. Although continuous release still provided slightly stronger fouling control, the advantage over the optimum intermittent mode was limited, whereas the energy benefit of interval operation was substantial. The present results, therefore, indicate that MNB-assisted hollow fiber ultrafiltration should be optimized on a membrane-specific basis and that transfer of interval settings from ceramic to polymeric systems without re-optimization is unlikely to be appropriate.

From an application perspective, intermittent MNB-assisted PVDF-HFM ultrafiltration is most relevant to low-pressure pretreatment processes, particularly as a pretreatment step before RO or NF systems in desalination and water reuse. In such integrated membrane trains, UF pretreatment is not intended for salt removal, but for reducing suspended solids, colloids, and organic foulants that can accelerate downstream RO/NF fouling. By mitigating organic deposition and improving cleaning recovery in PVDF-HFM, the proposed MNB strategy may help reduce the fouling load imposed on subsequent high-pressure membrane units.

However, the application boundary of this method should be recognized. Humic acid was used in this study as a representative model foulant for natural organic matter, which allowed controlled comparison among different MNB operating modes. Real wastewater or seawater contains more complex foulant matrices, including proteins, polysaccharides, biopolymers, colloids, microorganisms, oils, and inorganic scaling precursors. These components may interact with each other and form organic–inorganic or bio-organic composite fouling layers that are more compact and less reversible than HA fouling alone [25]. Therefore, the present results should be interpreted as a proof-of-concept demonstration under controlled organic fouling conditions. Further studies using real seawater, municipal wastewater effluent, and mixed-foulant feedwaters are needed to evaluate long-term stability, biofouling control, scaling tendency, and compatibility with downstream RO operation.

The choice between PVDF-HFM and ceramic membranes should also be application-specific. Polymeric membranes such as PVDF are generally more cost-effective and are suitable for conventional low-pressure UF applications under relatively mild water-quality conditions. By contrast, ceramic membranes may be justified when the feedwater or operating environment is chemically, thermally, or mechanically harsh, such as high-temperature streams, strongly acidic or alkaline wastewater, oily wastewater, industrial effluents containing abrasive particles, or systems requiring frequent intensive cleaning. Although ceramic membranes have higher initial costs, their superior chemical resistance, thermal stability, mechanical robustness, and longer service life may provide advantages from a life-cycle perspective. Thus, PVDF-HFM and ceramic membranes should not be viewed as directly interchangeable options; their selection should depend on feedwater characteristics, cleaning requirements, target lifetime, and overall life-cycle cost.

## 4. Conclusions

This study systematically evaluated the antifouling performance of MNB-assisted ultrafiltration using a PVDF-HFM with a focus on release pressure and intermittent aeration strategy. The results demonstrate that both bubble characteristics and temporal release mode govern membrane performance.

Increasing the release pressure to 0.60 MPa generated smaller and more concentrated MNBs, which effectively mitigated membrane fouling by enhancing bubble–foulant interactions and limiting foulant accumulation. Under this condition, a clear interval-dependent behavior was observed. Among the tested modes, a 15 min intermittent interval provided the optimal balance, achieving a rejection efficiency of 75% and a cleaning efficiency of 93%, which were close to those obtained under continuous operation. In contrast, prolonged intervals led to a rapid decline in fouling control, indicating that insufficient bubble replenishment weakens both turbulence-induced shear and interfacial protection.

A key finding of this work is that the optimal intermittent release interval is membrane-dependent. The PVDF-HFM exhibited a shorter optimal interval (15 min) than that reported for ceramic membranes (30 min), which is attributed to differences in surface wettability, structural flexibility, and local hydrodynamics that influence bubble retention and foulant detachment.

From an engineering perspective, intermittent MNB release reduced energy consumption by approximately 50% while maintaining comparable membrane performance. These findings highlight that membrane-specific optimization of MNB operation is essential for practical applications. The identification of an optimal intermittent window for PVDF hollow fiber ultrafiltration provides a basis for developing energy-efficient fouling control strategies in polymeric membrane systems.

## Figures and Tables

**Table 1:** Specifications of PVDF HNM.

Properties	Value
Filtration area (m^2^)	0.0064
Pore size (nm)	100
Contact angle (°)	56.21
Recommended operating flux (L m^−2^ h^−1^)	10–30
Operating pressure (MPa)	0.02–0.05

**Table 2:** Feed water parameter characterization.

Parameters	Value
pH	6.92~7
Temperature (°C)	22~28
UV350	0.375
Turbidity (NTU)	6.97
COD (mg L^−1^)	42
Conductivity (μs cm^−1^)	422.4

## Data Availability

The original contributions presented in this study are included in the article. Further inquiries can be directed to the corresponding author.
